# Biologically-Based Complementary and Alternative Medicine (CAM) Use in Cancer Patients: The Good, the Bad, the Misunderstood

**DOI:** 10.3389/fnut.2019.00196

**Published:** 2020-01-24

**Authors:** Kathryn Knecht, David Kinder, Amy Stockert

**Affiliations:** ^1^Loma Linda University School of Pharmacy, Loma Linda University, Loma Linda, CA, United States; ^2^Department of Pharmaceutical and Biomedical Sciences, Raabe College of Pharmacy, Ohio Northern University, Ada, OH, United States

**Keywords:** CAM, alternative medicine, anti-cancer, herbal, supplemental therapy

## Abstract

As complementary and alternative medicine (CAM) becomes more popular, it is being used in cancer patients to aid in recovery or to treat symptoms associated with the current chemotherapy. Numerous papers exist that discuss patients using CAM with cancer chemotherapy and their outcomes—both positive and negative. However, in the case of the negative outcomes, the reason for the dangers or interactions with drugs are not made clear. Indeed, many chemotherapy regimens are rendered less effective by the well-meaning but uninformed patient or their family members and friends. Similarly, reports of positive outcomes with CAM and chemotherapy provide a strong basis for further research, but do not identify specific mechanisms of action. These small clinical studies and *in vitro* studies identify a necessary area for further research and provide a much needed, although often rejected, alternative look at whole treatment plans. Careful review of the available information and evaluation of the nature of the CAM effects are necessary to combat the misunderstanding and sometimes unwarranted claims over CAM use. This mini review will explore some of the commonly used CAM agents and their mechanisms of interactions with other treatments. Suggestions as to which agents can be safe and when to use them will be an integral part of this review.

## Introduction

The use of Complementary and Alternative Medicine (CAM) is prevalent in the United States. Results of the 2012 National Health Institutes Survey show that 33.2% of adults in the US reported use of CAM therapies (1) with non-vitamin, non-mineral supplements as the most common modality (17.7%). Most use of CAM is complementary (2), meaning that patients add CAM to conventional therapies (2). Among cancer patients, CAM is also used with some variation due to cancer type and other factors; Davis et al. (3) summarized studies showing CAM use between 11 and 95% among cancer patients, though most studies estimate one third to one half (4–10).

## CAM Awareness

Knowledge accessibility has increased in recent years as has the patient's desire to participate in their health care. This desire is amplified for the cancer patient, who is faced with potentially life threatening news. The desire to try everything, and take a chance on what might work is overwhelmingly tempting even for patients with a significant medical background. One study found that the most prevalent reason for CAM use was “just trying to do everything that can help” (11, 12). Internet and social media provides an opportunity for patients to gain information and form opinions which guide their treatment decisions (13). Although many reputable resources exist, it is difficult for patient's to identify which sources provide peer-reviewed and scientifically sound reference. The desire to self-diagnose and self-treat grows as a result of the accessible knowledge and patient healthcare mistrust. Patients concerns over healthcare are in part born of the influence of social media. Susceptibility to medical conspiracy theories, distrust of the medical establishment, and preference for CAM are interrelated (14, 15); one of the six most common medical conspiracies is the belief that the Food and Drug Administration (FDA) is suppressing a natural cure for cancer. It is important that the media recognize their influence on healthcare decisions and inform the patients carefully.

It is also important that health care providers are aware of CAM use. Of concern is the fact that many patients, including cancer patients, do not communicate CAM use to their health care providers (3, 4, 16–18). In response to concerns of low disclosure, the National Center for Complementary and Alternative Medicine launched a “Time to Talk” campaign to encourage patient-provider communication about CAM (19). Disclosure is higher for women than for men and more likely in patients with a life-threatening illness (18). Furthermore, patients seek CAM information more from family, friends, and the internet than from health care providers (11, 20).

It may be a factor that there is in many cases a stigma placed on alternative therapies by the medical profession. A recent study evaluated healthcare providers' belief that they needed to be informed of a patient's CAM usage due to potential dangers. An overwhelming amount of the healthcare providers felt that this was essential knowledge and a patient must share it. Approximately 80% of the providers in the surveyed population cited that interactions with herbals and prescribed medications are problematic. Interestingly, only 15% of these healthcare providers were able to elaborate on the mechanism or specifics of the interaction, let alone the potential dangers (21). In some ways, this recognition of partially lacking knowledge on the part of the healthcare provider drives the CAM stigma. Recognizing their own knowledge gap only intensifies the providers' concerns, often leading to provider disregard for potential treatments. Patients recognize this disregard and opt out of reporting use of such products. These observations highlight a communication gap. Overcoming such a communication barrier with an open mind from both parties provides opportunities to evaluate the potential benefits of supplemental therapies in a safe and informed manner.

The purpose of this review is to consider not only the reports of adverse effects, both in the lab and in human populations, but also those studies demonstrating potential benefits. Evaluation of the available literature will focus on studies ranging from year 2010 to 2019 that describe the effects, both positive and negative, that address the efficacy and safety of CAM in cancer treatment. Although multiple modes of CAM exist, the focus will be on biologically-based CAM approaches, in particular dietary supplements.

## Adverse Effects

Potential adverse effects of CAM, both direct and indirect due to CAM-drug interaction, are an important concern in cancer treatment, and the Society for Integrative Oncology recommends routine evaluation of a patient's supplements for possible side effects and potential interactions (20, 22).

Reporting bias (23) in general could overstate the occurrence of adverse events due to the population failing to accurately report CAM usage when no adverse effects are noted. Similarly, patients experiencing adverse effects are more likely to report and more likely to identify their response as a CAM interaction. This phenomenon could abnormally skew the data to overestimate adverse reactions. Other studies emphasize the danger of mixing CAM with prescribed medicines. Although the potential for adverse reactions most definitely exist, it is difficult to estimate the true risk given limited reporting and essentially non-existent quality control over the CAM utilized, as well as the inherent difficulty in distinguishing disease symptoms, CAM effects, and effects of cancer treatment (7).

Peer-reviewed scientific evidence does exist for many herbal/supplemental remedies. Some evidence does support the proposed adverse reactions, and are reproducibly demonstrated in *in vitro* lab settings or in some cases animal models. These studies suggest the potential risk is significant enough that healthcare providers need to be aware of what their patients are using for self-treatment. However, despite the risk illuminated in these studies, only a few studies in the human population with accurate reporting confirm this level of risk. The vast majority of studies completed as a meta-analysis or retroactive study fail to support the grave nature of the proposed risks. It is important to consider that if dangers can be overstated based on self-reporting bias, the apparent lack of danger suggested by these studies may also be overstated.

## Possible Benefits

Biologically-based CAM (BB-CAM) approaches are used by cancer patients for a number of reasons, including cancer prevention; cancer treatment; strengthening of the immune system; improving well-being; and alleviating symptoms of either the disease or disease treatment, such as nausea, insomnia, and pain (7, 11). While numerous studies exist looking at the effects of supplements and herbals currently used as CAM on cell culture and or animal models, much less literature is available showing the long terms effects on patients using these much less on patients using them in combination with other therapies.

## Prevention

Evaluation of literature suggesting the preventative effects of these therapies are endless. Although these studies provide solid and reputable scientific method to evaluate the preventative potential of CAM, the same limitation always comes into play, i.e., the lack of long term controlled human trials, exists. This leaves virtually all of the valuable information variable in the real world population and therefore makes strong and confident recommendations difficult if not impossible. Table 1 provides a number of examples of CAMs with proposed cancer prevention abilities, Many studies demonstrate tissue specific or cell specific experimentation, which although beneficial in future research decisions, does not directly apply to the general population and makes generalization difficult. On the other hand, epidemiologic studies do suggest possible benefits in larger populations from dietary approaches [e.g., vegetarian (57, 58)], but do not establish cause and effect. A focus on dietary components, such as vitamins and minerals (Table 1) does indicate that deficiencies can introduce susceptibility and suggests repletion might be beneficial.

**Table 1:** Selected CAM approaches considered for the prevention and treatment of cancer.

**Category**	**CAM treatment**	**Effect/concerns**	**References**
Prevention	Antioxidants	Decreased cancer risk, food more than supplement	(24)
	Fiber	Decreased risk of colorectal and other cancers	(25)
	Folic acid	Increased, unchanged, and decreased risk of cancer	(26)
	Ginseng	Decreased risk of breast, colon, lung, and other cancers	(27)
	Grape seed extract	Antioxidant, decreased risk of cancer	(28)
	Green tea	Decreased risk of oral–digestive, prostate, lung, and other cancers	(29)
	Lycopene/tomato	Antioxidant, decreased risk of prostate cancer	(30, 31)
	Selenium	Decreased cancer risk if not selenium deficient, decreased recurrence	(32, 33)
	Turmeric/curcumin	Antioxidant, *in vitro* effects	(34)
	Vitamin C	Decreased risk of breast cancer recurrence	(35)
	Vitamin D	Increased, unchanged, and decreased risk of cancer depending on site, decreased recurrence	(36–40)
	Vitamin E	Decreased risk of breast cancer recurrence	(35)
Treatment	Antioxidants	Possible interference with chemotherapy, possible tissue protection	(41)
	Laetrile	Putative cancer treatment, no established benefit, risk of cyanide toxicity	(42)
	Milk thistle/silymarin	Chemotherapy adjunct, antioxidant, antiproliferative	(43)
	Mistletoe	Chemotherapy adjunct, benefits not established	(44)
	Omega-3 fatty acids	Possible chemotherapy enhancement	(45)
	Turmeric/curcumin	Chemotherapy adjunct, antioxidant	(34, 46)
	Vitamin C	High dose intravenous only	(47)
	Vitamin D	Improved response to immunotherapy in vitamin D-deficient patients	(48)
Immune support	Maitake	Potential immune stimulation and anti-cancer effects	(49)
	Reishi	Potential immune stimulation and anti-cancer effects	(50)
	Shiitake	Potential immune stimulation and anti-cancer effects	(51)
Symptomatic	Essiac tea	Quality of life, benefits not established	(52)
	Ginger	Nausea	(53, 54)
	Ginseng	Fatigue	(55)
	Marijuana	Nausea, pain	(56)
	Mistletoe	Quality of life, benefits not established	(44)
	Omega-3 fatty acids	Cachexia	(45)
	Selenium	Protection from radiation effects	(32)

## Treatment

In regard to treatment, many studies have explored anti-cancer mechanisms of specific supplement products. Overall mechanisms of anti-cancer activity are often reported as effects on a single biochemical target. However, a biochemical target could alter multiple pathways in the cell. Moreover, an individual chemical component of a supplement could influence more than one biochemical target. Finally, a supplement could contain more than one biologically active component. Changes can be cell-specific or in some cases epigenetically-determined and therefore patient-specific, precluding a generalizable recommendation. Genetics can thus play a significant role in patient response and/or toxicity. Attempts to understand all of these influences targets can be never-ending, and therefore make it impossible to describe a single definitive effect to medical professionals or patients. Even the single antioxidant lycopene, for example, has been reported to affect multiple targets including cell cycle progression, matrix metalloprotein activity (MMP-9), insulin-like growth factor (IGF), apoptosis, and TNF (59). Complexity and lack of a clear mechanism can contribute to the stigma of CAM as unscientific and unproven.

Numerous non-targets, or overall general influxes on the cells signaling mechanisms indeed complicates the problem. Complexities increased yet again considering the cancer cells already has a modified signaling sequence of events. These changes can be cell specific or in some cases epigenetically movies and therefore patient specific. This, the difficulty in applying a one size fits all recommendations for CAM is virtually impossible. This review is aimed at demonstrating this impossibility and reasserting focus on safety in human population and a scene of adverse effects by opening the line of communication between HCP and patient. In reality, the patient does not always expect or require a detailed understanding. Many times, while feeling hopeless and doubtful of traditional chemotherapeutic success, a patient may be eased simply hearing that there is no evidence that alternative treatments will harm them.

It should be noted that dietary modifications are often adopted by cancer patients (60). The benefits of most dietary modifications have not been established, but there is evidence that a ketogenic diet can be helpful as an adjunct in treatment of a number of cancers with minimal adverse effects (61–63). Attention to nutrition is of course important for general patient health, since nutritional deficiencies are common in cancer patients (64). However, correction of vitamin deficiency, vitamin D in particular, could not only benefit general health (65) but could enhance treatment (48). The importance of adequate nutrition can be agreed upon by patients and even the most skeptical of practitioners and could be an area of rapprochement.

## CAM-drug Interactions

A significant degree of complexity is added when considering the use of CAM as adjunct therapy. The majority of the studies showing benefit of CAM adjunctive therapy report improvements to patients' quality of life during chemotherapy rather than increased therapeutic efficacy. However, there are some studies that suggest efficacy may be increased with supplement usage. Increased survival rates for colon, gastric, and lung cancer were demonstrated with use of Pan-Asian medicine (PAM) (66–68) CAM, including supplement use, was also associated with improved quality of life (46, 69). Emotional distress, depression, insomnia, nausea, loss of appetite, and other symptoms as a result of disease or treatment can be addressed by CAM methods (see Table 1). Note that non-biologically-based CAM methods, such as meditation, deep breathing, or acupuncture can address some of these issues without risk of drug interactions and for this reason should be encouraged in CAM patients (20).

## Specific Adverse Effects

A major concern is the potential for adverse effects due to supplement-drug interactions, which could be either pharmacodynamic or pharmacokinetic.

Antioxidants neutralize the free radicals that can damage DNA and other cellular targets and can therefore be seen as protective. Increased dietary antioxidant intake is inversely associated with cancer occurrence, although antioxidant supplements have not shown equally beneficial effects (24). In regard to adjunctive therapy, antioxidants could interfere with radiation or with any chemotherapy that operates via a free radical mechanism (45). In regard to lung cancer, administration of vitamin A has even been found to correlate with worsened cancer progression (70, 71). Studies of antioxidant use in general have been mixed, but it may be appropriate to suspend antioxidant therapy 48 h before and after treatment sessions (20, 72). Interestingly, omega 3 fatty acids could enhance chemotherapeutic effects by increasing the free radical formation (45).

Anticoagulation is a possible adverse effect of some dietary supplements and could be an issue in patients suffering from blood dyscrasias or undergoing surgery (31, 72). Fish oil, ginger, garlic, green tea, curcumin/turmeric, and reishi are supplements used by cancer patients that could have anticoagulative effects (11, 72, 73).

Supplements, such as milk thistle (silybin), licorice, soy, black cohosh, and curcumin could have estrogenic effects and therefore are a concern in hormone-sensitive cancers (7, 72, 74). However, evidence of adverse effects in patients has not been established.

## Pharmacokinetic Concerns

Additionally, and substance that inhibits the breakdown of chemotherapeutics can increase Max concentrations of the drug and alter therapeutic intervals. Although these effects can increase drug efficacy, increased drug concentrations also lead to increased risk for toxicity. Conversely, the induction of drug metabolism can decrease drug concentrations and impair effectiveness. Effects on drug transport function similarly to effects on metabolism, altering movement of drugs into or out of the body or specific body compartments, however, many interactions are theoretical and based on *in vitro* rather than clinical studies (22, 72). In a cohort of 153 patients using both dietary supplements and chemotherapy, suspected drug interactions were found in 82 (54%) of patients but only one interaction was potentially clinical significant and no patient demonstrated adverse effects (22). Similarly, an analyses of 42 and 84 patients and found multiple theoretical interactions but no evidence of clinical consequences (7, 75). Nonetheless, the existence of potential interactions emphasizes the importance of good communication between patient and provider and the need for careful attention to dosing.

## Conclusions and Recommendations

Biologically-based and other CAM methods are a significant factor in the prevention and treatment of cancer for many patients, yet information on the safety and efficacy of these approaches are sadly lacking. *In vitro, in vivo*, and preliminary clinical studies indicate promise for some products in decreasing the growth of cancer cells, the progress of cancer, and the symptoms experienced by the patients, but their usefulness has not been established. Similarly, adverse effects including pharmacokinetic interactions with drugs are suspected in a number of cases, but the clinical impact is unknown. More research is needed, but in the meantime open communication between patients and providers is important in sharing what is known, avoiding potential hazards, and providing direction for future studies.

## Author Contributions

DK worked in the initial literature search and edits of the manuscript. KK added the referenced details and Table 1. AS completed the initial draft and finalized the submission.

### Conflict of Interest

The authors declare that the research was conducted in the absence of any commercial or financial relationships that could be construed as a potential conflict of interest.
